# Reply to “Survival analysis in a prediction model for early systemic recurrence in breast cancer”

**DOI:** 10.1002/cncr.34415

**Published:** 2022-08-15

**Authors:** Tomo Osako, Masaaki Matsuura, Hitoshi Tsuda, Shinzaburo Noguchi

**Affiliations:** ^1^ Division of Pathology Cancer Institute, Japanese Foundation for Cancer Research Tokyo Japan; ^2^ Division of Cancer Genomics Cancer Institute, Japanese Foundation for Cancer Research Tokyo Japan; ^3^ Department of Basic Pathology National Defense Medical College Saitama Japan; ^4^ Hyogo Prefectural Nishinomiya Hospital Hyogo Japan

We thank Qiu et al. for their interest in our study.^1^ Referring to the Transparent Reporting of a Multivariable Prediction Model for Individual Prognosis or Diagnosis statement,^2^ they remarked on four potentially insufficient descriptions in our article in terms of a prediction model study. The four limitations were as follows: patient follow‐up information, missing data handling methods, 95% confidence intervals (CIs) for the model performance, and a patient selection diagram. Two of these items were not described in the article; however, the remaining two items already were stated in the article or seemed unnecessary to include in the article.

Qiu et al. also mentioned a potential flaw in the multivariate analysis technique used in our study. However, the Cox model recommended by them could not be used to predict the probability of recurrence and validate the models in our study. In the following sections, we respond to each of the five items individually.

## PATIENT FOLLOW‐UP INFORMATION

Qiu et al. pointed out that the starting and ending times for follow‐up should have been mentioned in the Adjuvant Treatment and Follow‐Up section. However, the starting time of the patient follow‐up was already mentioned in the Patient Selection section (i.e., from 2008 to 2012), and the follow‐up period was included in the Patient Characteristics section (i.e., median follow‐up period, 5.5 years [range, 0.0–9.1 years]).

## MISSING DATA HANDLING METHODS

The authors stated that the methods for handling missing data should have been included in the article. Because of the strict word limit of the original article, we were unable to include the missing data handling methods. In our study, we used the pairwise deletion (available case analysis) technique for univariate analyses and the listwise deletion (complete case analysis) technique for multivariate analyses and internal validation.

## 95% CIs OF THE MODEL PERFORMANCE

The authors mentioned that the 95% CIs of the model performance (i.e., the sensitivity and the specificity) should have been included in the article. Although the 95% CIs of the model performance were missing from the article, these intervals could be calculated easily with the binominal Wald approximation method for Table 4 and with normal approximation for the 10‐fold cross‐validation results in Supporting Tables 6 and 7.^1^

The 95% CIs were calculated as follows: For the recurrence‐related model, the average sensitivity, specificity, and accuracy were 73.5% (95% CI, 61.9%–85.1%), 77.0% (95% CI, 68.0%–86.0%), and 76.9% (95% CI, 68.6%–85.2%), respectively, for the first internal validation using the training cohort with the 10‐fold cross‐validation procedure and 61.9% (95% CI, 47.2%–76.6%), 80.0% (95% CI, 77.9%–82.0%), and 79.5% (95% CI, 77.4%–81.5%), respectively, for the second internal validation using the validation cohort. For the recurrence prediction model, the average sensitivity, specificity, and accuracy were 81.5% (95% CI, 66.9%–96.2%), 70.0% (95% CI, 55.7%–84.2%), and 70.5% (95% CI, 57.4%–83.5%), respectively, for the first internal validation and 63.4% (95% CI, 47.2%–80.0%), 81.7% (95% CI, 79.3%–84.0%), and 81.1% (95% CI, 78.8%–83.5%), respectively, for the second internal validation.

## PATIENT SELECTION DIAGRAM

Qiu et al. recommended that we present a patient selection diagram in our article. However, the patient selection procedure, including the inclusion and exclusion criteria, was included in the Patient Selection and Patient Characteristics sections of our article. Therefore, we did not draw a diagram because there was no additional information.

## MULTIVARIATE ANALYSIS TECHNIQUE

Qiu et al. recommended using the Cox regression model instead of the logistic regression model in our study. However, the logistic model was an appropriate statistical method for our study, whereas the Cox model could not accomplish the purposes of our study.

The Cox model can analyze censored data; however, it cannot calculate individuals' estimated probabilities, which are essential for model validation. In contrast, the logistic model can calculate the estimated probabilities of individuals. The main objectives of this study were to establish the predictive models with the training and validation cohorts and to publish the online tool for estimating the probability of 5‐year distant recurrence–free survival rates (https://www.theranostics.jp/sln/show). Therefore, we constructed the predictive models with logistic regression analysis.

## CONFLICTS OF INTEREST

Tomo Osako has received honoraria from Diaceutics and consulting fees from Chiba Cytopathology Laboratory outside the submitted work. Hitoshi Tsuda has received research funding from Taiho, Goryo Chemical, and Roche Diagnostics and scholarship donations from Chugai, Takeda, and Eli Lilly outside the submitted work. Shinzaburo Noguchi is an advisor to Sysmex and has received consulting fees and research funding from Sysmex; he is an advisor to AstraZeneca and Nittobo and has received honoraria and/or consulting fees from AstraZeneca, Eli Lilly, Chugai, Nittobo, and Sysmex outside the submitted work; and he holds joint patents not related to this study with Sysmex. The other author made no disclosures.

## FUNDING INFORMATION

Sysmex.
